# Topics of Public Interest

**Published:** 1910-07

**Authors:** 


### TOPICS OF PUBLIC INTEREST.

### Appointments by Regents

### Reciprocal Relations with Jersey on Medical Schools Annulled.

THE Regents of the University of the State of New York, at the regular meeting of the board held at Albany, June 9, 1910, made the following appointments:

William Warren Potter and Lee H. Smith, Buffalo, and Glentworth Reeve Butler, Brooklyn, as members of the state board of medical examiners; James Law, Ithaca, F. C. Greenside, New York; R. C. Reed, Elmira; H. S. Beebe, Albion; and George A. Knapp, Millbrook, members of the state board of veterinary examiners; A. M. Holmes and H. J. Burkhart, reappointed, and E. G. Parker, Poughkeepsie, members of the state board of dental examiners; Charles F. Prentice, New York, and George R. Fox, members of the state board of examiners in optometry; Miss Lydia B. Anderson, reappointed, to serve on board of nurse examiners; Charles S. McCulloch, Leon Orr Fisher and Samuel D. Patterson, reappointed members of the state board of certified public accountants.

Warren L. Bradt, secretary of the present board of pharmacy, was appointed secretary of the new board at a salary of $3,000 a year.

The board confirmed the nomination of Dr. Charles F. Wheelock, chief of the examinations division of the State Education Department, as second assistant state commissioner of education, to succeed Dr. Frank Rollins, resigned.

The resignation of Miss Anna L. Alline as inspector of nurse training schools was accepted, and Miss Annie W. Goodrich, of New York, appointed to succeed her.

The appointment of Arnold J. F. Van Laer, archivist, to translate and publish the Dutch manuscript records of the state, was confirmed.

Owing to the recent lowering of the standards of preliminary education required for admission to medical schools by New Jersey below the standard required for admission to medical schools in this state, the board annulled the reciprocal relations between New York and New Jersey.

The Regents' rules in relation to commercial schools and commercial subjects were amended.

The medical license of Charles Francis Baxter was revoked and his registration cancelled. Baxter was convicted in Suffolk County of frauds in connection with insurance papers.

---

### State Board of Pharmacy will now be Reorganized

Governor Hughes signed the bill June 8, 1910, of Assemblyman Whitney, reorganising the state board of pharmacy. It provides for the appointment by the state board of regents of a board of nine examiners to replace the fifteen now in office. The bill also makes provision for a standard of drugs and medicines and provides penalties for selling adulterated and misbranded drugs.

---

### Open Air School

**Aldermen appropriate $1,200 to equip Two Rooms—One to be Kitchen—Twenty Children will be selected for this Initial Test.**

Acting upon the report of a committee of the Buffalo Association for the Relief and Control of Tuberculosis, Superintendent Emerson of the school department recently requested an appropriation of $1,200 to equip two open-air schoolrooms. The request was granted by the aldermen according to the *Buffalo Express,* June 14, 1910.

One of the rooms will be fitted up for the preparation of food for the children. To start with twenty pupils will be selected by the medical school inspectors.

In its report upon this feature of education the committee of the tuberculosis commission stated that the effect of proper breathing, pure air, plenty of exercise, and proper food would be found so beneficial in the case of puny children, it would be apparent that fresh air in abundance could not fail to be of advantage to healthy children, and the result will lead to a demand for the proper construction of school buildings. The children undergoing the test are to be weighed and measured each week.

---

"Doctor, I'm all run down and extremely nervous. Can you save me?"

"Surely, my friend; surely. Yours is a common ailment just now. You are simply reading more baseball news than you can assimilate."—*Washington Herald.*

